# Impact of awake prone positioning duration on intubation or mortality in COVID-19 patients with acute respiratory failure: secondary analysis of a randomized clinical trial

**DOI:** 10.1186/s13613-025-01501-8

**Published:** 2025-06-23

**Authors:** Qin Sun, Rui Zhang, Junyi Zhang, Jianfeng Xie, Yingzi Huang, Yi Yang, Haibo Qiu, Ling Liu, Hui Chen, the Chi-ARDS Net (Chinese ARDS Research Network)

**Affiliations:** Jiangsu Provincial Key Laboratory of Critical Care Medicine, Department of Critical Care Medicine, Zhongda Hospital, School of Medicine, Southeast University, 87 Dingjiaqiao Rd., Nanjing, 210009 People’s Republic of China

**Keywords:** COVID-19, Prolonged awake prone positioning, Intubation, Mortality

## Abstract

**Background:**

Compared with shorter awake prone positioning (APP), prolonged APP (≥ 12 h daily) reduces the intubation rate in patients with COVID-19-related acute hypoxemic respiratory failure (AHRF). However, the optimal APP duration is uncertain. In this secondary analysis, we aimed to explore whether a longer APP duration is associated with improved outcomes and to identify the optimal duration of APP.

**Methods:**

Data from a multicenter randomized controlled trial involving nonintubated COVID-19 patients with AHRF were analyzed. Daily APP duration over 7 days after randomization was recorded as the primary exposure in present study. The primary outcome was the time from randomization to APP failure, which was defined as a composite of tracheal intubation or mortality within 28 days. A Cox proportional hazards regression model was employed to elucidate the associations, and the daily duration of APP was treated as time dependent.

**Results:**

A total of 409 patients were randomized in the original trial, and 408 were enrolled in this analysis. Among these patients, 105 (25.7%) experienced APP failure. A longer daily APP duration was associated with a lower risk of APP failure, with a hazard ratio (HR) of 0.93 (95% confidence interval (CI): 0.88–0.98), and the association was significant only during the first three days after randomization. There was a nonlinear relationship between the daily APP duration and the risk of APP failure (P = 0.015 for nonlinearity). Compared with patients whose APP duration ranged from 8 to 12 h per day, patients with less than 8 h of APP per day had a greater risk of APP failure (HR 2.44, 95% CI 1.21–4.92), whereas extending APP beyond 12 h per day did not improve the outcomes further (HR 1.03, 95% CI 0.51–2.10, P = 0.932).

**Interpretation:**

A longer daily APP duration was associated with a reduced risk of APP failure in COVID-19-related AHRF patients, and the optimal APP duration was 8–12 h per day.

*Clinical trial* ClinicalTrials.gov: NCT05677984, Registered January 3, 2023. https://register.clinicaltrials.gov/prs/app/action/SelectProtocol?sid=S000CST9&selectaction=Edit&uid=U0000YKY&ts=4&cx=-x0muek

**Supplementary Information:**

The online version contains supplementary material available at 10.1186/s13613-025-01501-8.

## Background

Awake prone positioning (APP), which involves placing non-intubated patients with acute hypoxemic respiratory failure (AHRF), chronic obstructive pulmonary disease (COPD), or any postoperative pulmonary complications in the prone position [1–3], can improve oxygenation and reduce intubation rates, particularly observed in COVID-19 patients with AHRF [4–9]. Consequently, the implementation of APP has been suggested in recently guidelines and swiftly integrated into the clinical management of COVID-19 patients with AHRF [10].

The therapeutic effects of APP have been shown to have a dose‒response relationship. A meta-analysis involving 17 trials revealed that a median duration of APP greater than or equal to 5 h per day significantly decreased the risk of endotracheal intubation, whereas durations shorter than 5 h per day did not confer that benefit [11]. Similarly, Esperatti et al. performed a cohort study showing a progressively decreasing odds ratio for intubation or mortality with increased duration in the prone position (measured in hours per day), with an APP duration of 16 h or more per day being associated with the lowest odds ratio for intubation or mortality [12]. However, none of these studies have elucidated the impact of APP duration as a continuous variable on outcomes in COVID-19-related AHRF patients, making it difficult to determine the optimal duration of APP. Additionally, APP is a dynamic therapeutic process that needs to be conducted daily, and the daily treatment effect of APP should be considered.

Liu and colleagues performed a multicenter randomized controlled study comparing prolonged APP and shorter APP on intubation within 28 days in COVID-19-related AHRF patients. The results demonstrated that compared with standard care (with a median APP duration of 5 h per day), prolonged APP (with a median APP duration of 12 h per day) reduced the intubation rate within 28 days [13]. However, that study did not clarify the precise relationship between daily APP exposure time and the intubation rate or mortality. Therefore, in this secondary analysis, we treated APP duration within the first seven days as a time-varying variable and aimed to clarify the relationship between daily APP duration and the intubation rate or mortality rate and to identify the optimal APP duration for COVID-19-related AHRF patients.

## Methods

### Study design and participants

This secondary analysis used data from the prolonged APP trial (ClinicalTrials.gov: NCT05677984), which was conducted in accordance with the Strengthening the Reporting of Observational Studies in Epidemiology (STROBE) statement. The prolonged APP trial was a multicenter, randomized controlled trial, and the protocol and results have been published. The trial was approved by the local ethics committee at participating sites. Written informed consent was obtained from the patients or their next-of kin before randomization. The present analysis was not prespecified in the original trial protocol.

The prolonged APP trial included nonintubated patients with COVID-19-related AHRF from 12 hospitals. The specific inclusion and exclusion criteria, as well as the study protocol, can be found in our previously published study [13]. Patients were randomly assigned to prolonged APP (target > 12 h daily for 7 days) or standard care with a shorter duration of APP. In this secondary analysis, all the patients enrolled in the prolonged APP trial were considered for inclusion. Patients with no data on APP duration were excluded from the study.

### Data collection and exposures

Details of the data collection can be found in the original trial. Patients in both arms of the previous RCT and their APP duration time were collected and analyzed. The primary exposure was the daily duration of APP, which was collected from the start of the day after randomization until intubation, death, the resolution of AHRF (SpO_2_ ≥ 93% on room air) or 7 days after randomization, whichever occurred first. The secondary exposures were the total duration of APP and the average duration of APP.

### Outcomes

The primary outcome of the study was the time from randomization to APP failure, which was defined as a composite of tracheal intubation or mortality within 28 days. The secondary outcomes (all censored at 28 days after enrollment) included the time from randomization to death, time from randomization to intubation, and time from randomization to hospital discharge.

### Statistical analysis

The values are presented as the means (standard deviations) or medians [interquartile ranges (IQRs)] for continuous variables, as appropriate, and categorical variables are presented as total numbers and percentages. Comparisons between groups were made with the X^2^ test or Fisher’s exact test for categorical variables and Student’s t test or the Mann‒Whitney U test for continuous variables, as appropriate. A P value < 0.05 was considered statistically significant when comparing differences. All the statistical analyses were performed with R (version 4.1.3, R Core Team, R Foundation for Statistical Computing, Vienna, Austria). The study schedule is presented in Figure S1.

We first employed a Cox proportional hazards regression model to estimate the effects of the primary and secondary exposures on APP failure, with the primary exposure (daily duration of APP) treated as a time-dependent exposure in the model. The hazard ratio (HR) and its 95% confidence interval was calculated. Baseline variables were purposefully selected as confounders and included age, sex, body mass index (BMI), location at enrollment, respiratory support with a high-flow nasal cannula or noninvasive ventilation, SpO_2_/FiO_2_, sequential organ failure assessment (SOFA) score and treatment assignment (prolonged APP and standard care). The E values were calculated to quantify the effects of unmeasured confounders (https://www.evalue-calculator.com/evalue/).

Restricted cubic splines (RCS) were also used to visualize the relationship between the exposures and APP failure. We subsequently transformed the continuous parameter of the daily APP duration into a dichotomized variable on the basis of the threshold obtained by RCS and included it in the Cox regression model. RCS was performed with the “rms” package (https://github.com/harrelfe/rms).

Similarly, the time to death was analyzed with a Cox proportional hazards regression model, with patients censored at 28 days after randomization. We created multivariable competing-risk regression models for both the time to intubation and the time to hospital discharge, where death prior to intubation or discharge was a competing event and intubation or hospital discharge was the event of interest, with the “dynamicLM” package in R (https://github.com/thehanlab/dynamicLM).

Three sensitivity analyses were performed. A Cox regression model with Heaviside functions was used to assess how the HR for APP failure changed over different exposure windows (1–3 days, 4–5 days, and 6–7 days); we also duplicated the main analyses after excluding patients who were intubated or deceased on Day 1. Finally, the impacts of daily APP duration on early APP failure (within 7 days) and late APP failure (beyond 7 days) were investigated separately. Several subgroup analyses were performed with stratification according to age, sex, admission type, baseline oxygen model and oxygenation.

## Results

### Baseline characteristics

**Fig. 1:**
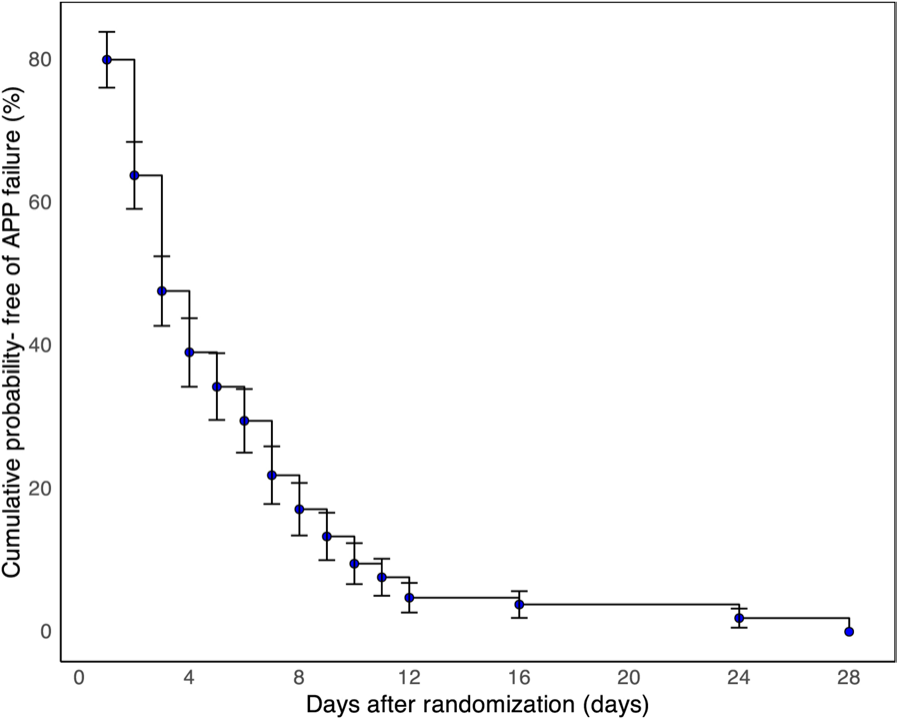
Kaplan–Meier plots showing the cumulative probability of APP failure

After one patient with no APP duration data was excluded, 408 patients were included in the secondary analyses. In the study cohort, 105 (25.7%) patients experienced APP failure within 28 days after randomization. The cumulative risk of avoiding APP failure is represented in Fig. 1. The baseline characteristics of and pharmacological interventions in patients who experienced failure and success with APP are shown in Table 1.

**Table 1:** Clinical characteristics between patients who failed and who succeeded on APP

	APP success (n = 303)	APP failure (n = 105)	P value
Age, year, median [IQR]	71 (63, 75)	71 (67, 77)	0.035
Sex (Male), No. (%)	212 (70.0)	70 (66.7)	0.611
BMI, kg/m^2^, median [IQR]	23.9 (21.8, 26.0)	24.2 (21.3, 26.0)	0.54
Location at enrolment, No. (%)			
Intensive care unit	13 (4.3)	24 (22.9)	< 0.001
Sub intensive care unit	61 (20.1)	44 (41.9)	
General ward	229 (75.6)	37 (35.2)	
Coexisting illness, No. (%)			0.703
Hypertension	156 (51.5)	57 (54.2)	0.703
Diabetes	99 (32.7)	39 (37.1)	0.475
Chronic heart disease	9 (3.0)	12 (11.4)	0.002
Chronic lung disease	6 (2.0)	4 (3.8)	0.289
Chronic kidney disease	20 (6.7)	7 (6.7)	1
Oxygenation mode, No. (%)			
Conventional oxygen therapy	229 (75.6)	33 (31.4)	< 0.001
High-flow nasal cannula	60 (19.8)	49 (46.7)	
Non-invasive ventilation	14 (4.6)	23 (21.9)	
SOFA score, median [IQR]	3 (2, 4)	4 (3, 6)	< 0.001
SpO_2/_FiO_2_, median [IQR]	240.0 (207.8, 322.4)	225.0 (177.4, 313.8)	0.003
FiO_2_, median [IQR]	37 (29, 45)	41 (29, 50)	0.002
Allocated to prolonged APP group, No. (%)	162 (53)	42 (40)	0.024
Time from hospital admission to randomization, days, median [IQR]	1 (0, 3)	1 (0, 2)	0.48
Pharmacological intervention, No. (%)			
Anti COVID-19	176 (58.1)	69 (65.7)	0.208
Steroids	181 (59.7)	73 (69.5)	0.096
Anticoagulants	255 (84.2)	88 (83.8)	1
Antibiotics	232 (76.6)	93 (88.6)	0.013

APP: Awake prone positioning; IQR: Interquartile range; BMI: Body mass index; SOFA: Sequential organ failure assessment; SpO_2_: Peripheral oxygen saturation; FiO_2_: Fraction of inspired oxygen

Compared with patients who were successful with APP, those who experienced APP failure were older, had higher SOFA scores, had lower SpO_2_/FiO_2_ ratios, had a lower likelihood of having received prolonged APP, and were more likely to receive HFNC therapy or noninvasive ventilation. The use of pharmacological interventions was fairly comparable between the groups. The average daily APP duration in the APP success and failure groups was 11.6 [IQR: 5.2, 13.3] and 6.1 [IQR: 3.0, 12.0] hours, respectively. Figure S2 shows that the daily duration of APP was consistently longer across the 7 days after randomization in the APP success group than in the failure group.

### Primary outcome

**Fig. 2:**
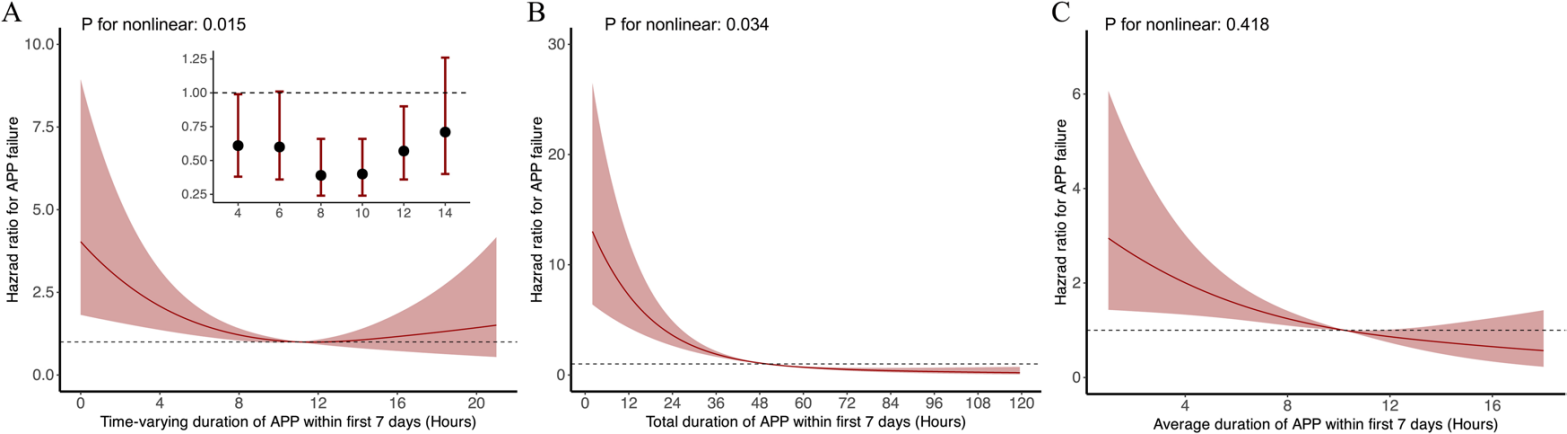
Association between APP duration and APP failure. **A** Adjusted relationship between time-varying duration of APP within first 7 days and APP failure; **B** Adjusted relationship between total duration of APP within first 7 days and APP failure; **C** Adjusted relationship between average duration of APP within first 7 days and APP failure. APP: Awake prone positioning

After adjustment, the multivariate Cox regression model demonstrated that daily APP duration was associated with a lower risk of APP failure, with an HR of 0.93 (95% CI: 0.88–0.98, P < 0.001) (Table 2 and Table S2). The result was robust unless there was an unmeasured confounder that was associated with a higher risk of APP failure with an HR greater than 1.28 (Figure S3A). Figure 2A shows that the daily APP duration had a nonlinear relationship with the probability of APP failure (P = 0.015 for nonlinearity). We further defined three categories of daily APP duration on the basis of the thresholds obtained by RCS; compared with patients with an APP duration ranging from 8 to 12 h per day, those with an APP duration less than 8 h per day presented a greater risk of APP failure (HR 2.44, 95% CI 1.21–4.92, P = 0.013), whereas extending the APP duration beyond 12 h per day did not further enhance the patient outcomes (HR 1.03, 95% CI 0.51–2.10, P = 0.932) (Table S1).

**Table 2:** Cox proportional hazard model to analyze the effect of the duration of APP on the primary outcome

	Primary exposure^#^		Secondary exposures^#^			
	HR (95% CI)	P value	HR (95% CI)	P value	HR (95% CI)	P value
Daily duration of APP, h	0.93 (0.88–0.98)	0.006	–	–	–	–
Total duration of APP, h	–	–	0.96 (0.95–0.97)	< 0.001	–	–
Average duration of APP, h	–	–	–	–	0.91 (0.86–0.96)	< 0.001
Age, years	1.03 (1.01–1.06)	0.005	1.02 (0.99–1.04)	0.122	1.03 (1.01–1.06)	0.009
Sex (Female)	1.05 (0.67–1.64)	0.835	1.06 (0.69–1.64)	0.791	1.06 (0.69–1.62)	0.787
BMI, kg/m^2^	0.99 (0.94–1.05)	0.792	0.97 (0.91–1.03)	0.336	0.99 (0.94–1.05)	0.821
Location at enrolment						
Intensive care unit	Reference	–	Reference	–	Reference	–
Sub intensive care unit	0.73 (0.43–1.23)	0.234	0.83 (0.50–1.38)	0.481	0.78 (0.46–1.31)	0.341
General ward	1.24 (0.33–4.61)	0.749	0.94 (0.34–2.61)	0.902	1.29 (0.46–3.64)	0.629
Non-invasive respiratory support^*^	6.04 (1.68–21.78)	0.006	5.00 (1.81–13.83)	0.001	6.12 (2.22–16.87)	< 0.001
SpO_2_/FiO_2_, per 10	0.88 (0.45–1.72)	0.699	0.89 (0.47–1.68)	0.720	0.85 (0.45–1.61)	0.612
SOFA score	1.28 (1.17–1.40)	< 0.001	1.23 (1.12–1.35)	< 0.001	1.27 (1.16–1.39)	< 0.001
Allocated to standard care group	0.97 (0.58–1.62)	0.901	0.36 (0.23–0.58)	< 0.001	0.81 (0.46–1.40)	0.447

*Non-invasive respiratory support including high-flow nasal cannula and non-invasive ventilation

^#^Primary exposure: The primary exposure was the daily duration of APP. Secondary exposures: The secondary exposures were the total duration of APP and the average duration of APP

APP: Awake prone positioning; HR: Hazard ratio; CI: Confidence interval; BMI: Body mass index; SpO_2_: Peripheral oxygen saturation; FiO_2_: Fraction of inspired oxygen; SOFA: Sequential organ failure assessment

For the secondary exposures, both the cumulative APP duration (HR 0.96, 95% CI 0.95–0.97, P < 0.001) and the daily average duration of APP (HR 0.91, 95% CI 0.86–0.96, P < 0.001) within the first 7 days were significantly associated with APP failure (Table 2, Table S3-S4 and Fig. 2). The E values were 1.2 and 1.34, respectively (Figure S3).

### Secondary outcomes

Ninety-one (22.3%) patients were intubated, and 93 (22.8%) patients died within 28 days after randomization. A longer daily duration of APP showed a marginally protective association with 28-day mortality (HR 0.95, 95% CI 0.91–1.003; P = 0.065) (Table S5). According to the competing-risk regression models, a longer daily duration of APP was associated with a lower risk of intubation (HR 0.86, 95% CI 0.75–0.99; P = 0.032) but had no effect on hospital discharge (HR 1.02, 95% CI 0.99–1.05; P = 0.13).

### Sensitivity and subgroup analyses

Using Heaviside functions, the association between the daily duration of APP and APP failure was found to be significant only during the first three days after randomization (Table S6). After patients who were intubated or died on Day 1 were excluded, the results were similar to those of the main analysis (HR 0.94, 95% CI 0.88–0.99; P = 0.029) (Table S7). Additionally, the effect of daily APP duration was unrelated to the timing of APP failure (Table S8).

**Fig. 3:**
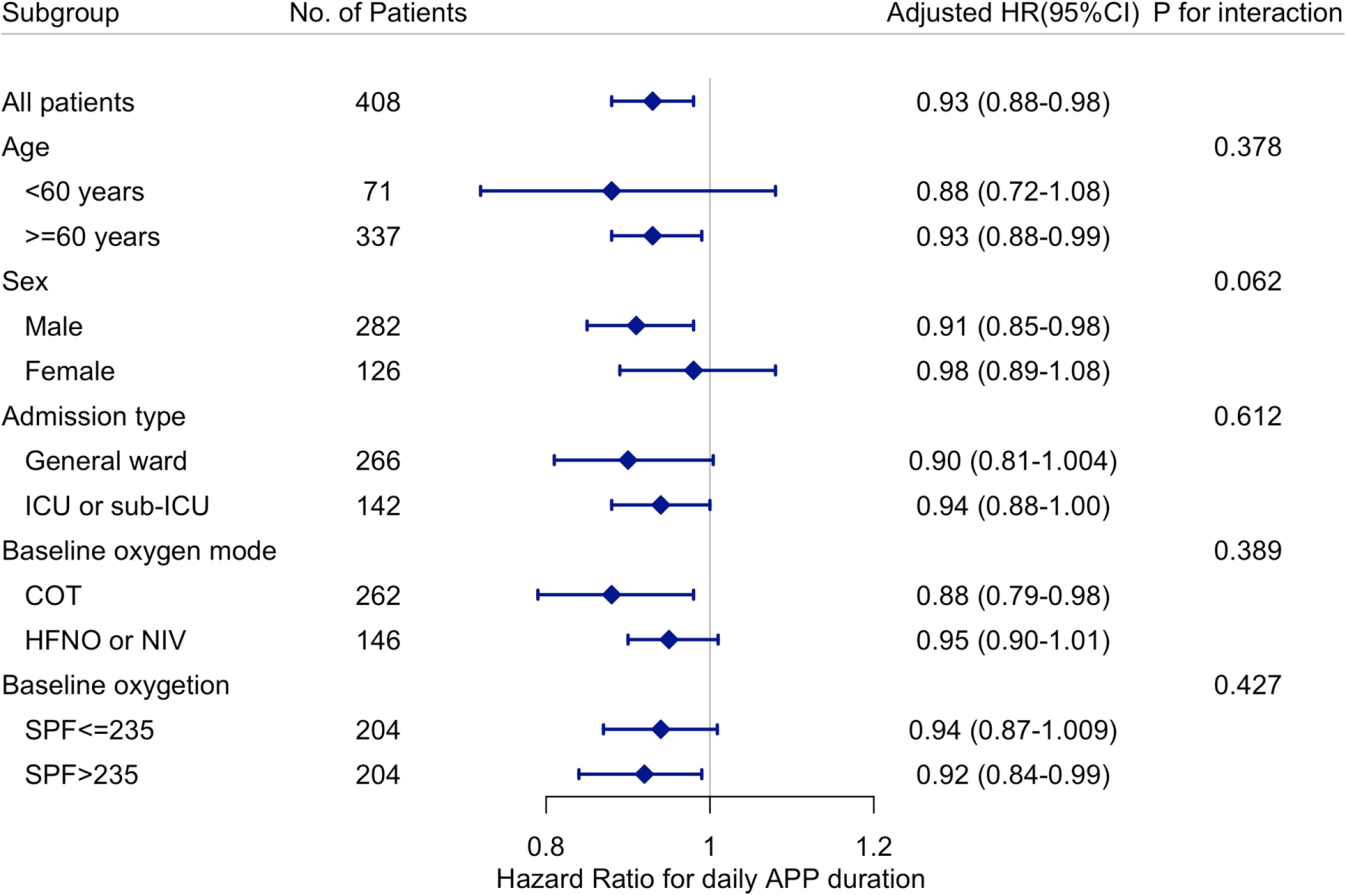
Subgroup analyses of association between daily APP duration and APP failure

According to the subgroup analyses (Fig. 3), the association between daily APP duration and APP failure appeared to be more pronounced among patients who were older (aged ≥ 60 years), were male, and received conventional oxygenation therapy, while no significant interactions were observed.

## Discussion

In this secondary analysis of a randomized controlled trial involving patients with COVID-19-related AHRF, we identified a nonlinear association between the daily APP duration and APP failure. A longer APP was associated with a reduced risk of intubation or death within 28 days, with the most significant effect observed during the first three days post-randomization. Notably, the protective effect of APP against intubation or mortality was the greatest when the APP duration was 8–12 h per day.

Prone positioning is considered a time-dependent treatment, and recent meta-analyses and reviews have suggested that the daily duration of prone positioning is a critical factor in determining its effectiveness [14–16]. However, previous studies reported that patients spend limited time in the awake prone position each day, which somewhat constrains the dose‒response analysis of the relationship between prone positioning duration and therapeutic outcomes [7, 8, 17]. In our study, patients received prolonged APP, and the APP duration ranged from 0 to 21 h per day, which was significantly longer than the durations reported in previous studies. Our results highlighted a strong nonlinear relationship between the APP duration and clinical outcomes for COVID-19 patients with AHRF. This is consistent with the findings in Mariano and colleagues’ research, which showed a strong association between extended durations of awake prone positioning and improved treatment outcomes [11]. Notably, our data were derived from an RCT, ensuring higher data quality, and by considering APP time as a continuous variable and incorporating dynamic factors, we determined the optimal duration of APP.

Implementing the optimal duration of APP is crucial; however, significant inconsistency exists in its application [18]. Although several physiological studies have shown that three hours or less in the awake prone position enhances oxygenation and optimizes the ventilation‒perfusion ratio [5, 19], early studies that focused on shorter APP durations reported no decrease in either the intubation rate or in-hospital mortality [20]. Ehrmann’s data revealed an increase in the proportion of treatment success with APP durations longer than 8 h [7]. A recent study also revealed that in patients with COVID-19 and hypoxemic respiratory failure, maintaining awake prone positioning for more than 8 h daily reduced the 28-day mortality rate. Mariano’s multicenter prospective cohort study even suggested that ≥ 8 h/d may be the duration necessary to reduce the risk of death, indicating that 8 h is an important threshold [11, 21]. Our findings suggested that 8 to 12 h per day in the awake prone position was the optimal duration; extending prone positioning beyond this timeframe did not yield additional clinical benefits. These findings were consistent with the findings of a recent meta-analysis of individual data, which found that an optimal duration of > 10 h during the first three days was associated with an increase in survival without intubation [22]. Two possible reasons might explain this: 1. The effect of prone positioning may reach a plateau, as previous physiological studies have shown that the improvement in oxygenation and the ventilation‒perfusion ratio achieved by prone positioning ventilation in mechanically ventilated patients does not further increase with prolonged prone positioning [23]. This pathophysiological phenomenon may also occur with awake prone positioning. 2. In this study, nearly all patients underwent prolonged APP, whereas the control group experienced a shorter duration of awake prone positioning [13]. The lack of a control group with no prone positioning treatment may have weakened the time-dependent effect of APP.

The timing of APP initiation in COVID-19-related AHRF patients has been a topic of considerable interest in clinical management. The evidence supports the benefits of the early initiation of prone positioning with regard to enhancing oxygenation and improving patient outcomes in intubated individuals with moderate to severe ARDS [24]. A case series study demonstrated the effect of early APP on improving oxygenation [25], and a later post hoc analysis of data collected for a randomized controlled trial further revealed that, compared with late APP, early APP (defined as APP initiated within 24 h of HFNC start) improved patients’ 28-day survival [26]. Our results demonstrated that the association between the daily duration of APP and APP failure was significant only within the first three days following randomization, which aligns with the principles proposed by Kaur and colleagues with regard to the early initiation of APP [26]. Although there may be differences from traditional ARDS, early pathophysiological changes in hypoxic respiratory failure due to COVID-19 still involve inflammation and damage to the alveolar epithelium, leading to impaired gas exchange, pulmonary edema, and reduced lung compliance [27]. The early initiation of APP during the exudative/inflammatory phase may have enhanced oxygenation, reduced the work required to breathe, and potentially improved the clinical outcomes.

This study has several notable strengths. It drew on data from a multicenter randomized controlled trial, ensuring the robustness and quality of the dataset. Furthermore, unlike prior studies, this research captured a wider spectrum of APP durations, enabling a more thorough and nuanced analysis of the association between APP duration and clinical outcomes in patients with COVID-19.

Several limitations should be noted. First, this analysis was secondary in nature and was not prespecified in the original protocol. The current analysis provides valuable exploratory insights but should be interpreted with caution due to its retrospective design and secondary nature. Second, both measured and unmeasured confounders were present in this study. Using the E value, we found that unmeasured confounders alone are unlikely to account for the observed treatment effect. Third, we did not collect certain data that may influence the interpretation of APP duration and APP failure. Specifically, information regarding the indication for intubation, the patient’s body position at the time of intubation (supine, prone, or lateral), and the time interval between the last positional change and intubation was not available. Fourth, while a daily APP duration of 8–12 h may be optimal for COVID-19 patients with AHRF, the generalizability of these findings is uncertain and needs further prospective validation. Additionally, the physiological mechanisms underlying the time-dependent effects of APP are not fully understood and require further investigation. Fifth, although we used a multivariable Cox regression with daily APP duration as a time-dependent variable to capture daily effects, the model did not adjust for censoring due to death or discharge. Finally, given the relatively mild respiratory failure in this study population, it is unclear whether the conclusions can be applied to all COVID-19 patients with AHRF. The generalizability of our findings to broader AHRF populations requires prospective validation.

In conclusion, there was a nonlinear relationship between daily APP duration and the intubation or mortality rate within 28 days in COVID-19-related AHRF patients, and a longer daily APP duration was associated with a reduced risk of APP failure. The association between the daily duration of APP and APP failure was significant only within the first three days following randomization. The optimal APP duration in this secondary analysis was 8–12 h per day. These findings support the early initiation of APP and provide a basis for recommendations about the optimal daily APP duration in nonintubated COVID-19 patients with AHRF.

## Supplementary Information

Additional file 1

## Data Availability

The data that support the findings of this study belong to the research project and access to the data can be obtained upon approval from the data management committee of the awake prone positioning research.

## References

[1] Verma AA, Razak F, Munshi L, et al. Awake prone positioning and covid-19. BMJ. 2022;379: o2888. 36740852 10.1136/bmj.o2888

[2] Koeckerling D, Barker J, Mudalige NL, et al. Awake prone positioning in COVID-19. Thorax. 2020;75(10):833–4. 32546571 10.1136/thoraxjnl-2020-215133

[3] Pavlov I, Li J, Kharat A, et al. Awake prone positioning in acute hypoxaemic respiratory failure: an international expert guidance. J Crit Care. 2023;78: 154401. 37639921 10.1016/j.jcrc.2023.154401

[4] Sun Q, Qiu H, Huang M, et al. Lower mortality of COVID-19 by early recognition and intervention: experience from Jiangsu Province. Ann Intensive Care. 2020;10(1):33. 32189136 10.1186/s13613-020-00650-2 PMC7080931

[5] Coppo A, Bellani G, Winterton D, et al. Feasibility and physiological effects of prone positioning in non-intubated patients with acute respiratory failure due to COVID-19 (PRON-COVID): a prospective cohort study. Lancet Respir Med. 2020;8:765–74. 32569585 10.1016/S2213-2600(20)30268-X PMC7304954

[6] Prud’homme E, Trigui Y, Elharrar X, et al. Effect of prone positioning on the respiratory support of nonintubated patients with coronavirus disease 2019 and acute hypoxemic respiratory failure: a retrospective matching cohort study. Chest. 2021;160:85–8. 33516704 10.1016/j.chest.2021.01.048 PMC7844382

[7] Ehrmann S, Li J, Ibarra-Estrada M, et al. Awake prone positioning for COVID-19 acute hypoxaemic respiratory failure: a randomised, controlled, multinational, open-label meta-trial. Lancet Respir Med. 2021;9(12):1387–95. 34425070 10.1016/S2213-2600(21)00356-8 PMC8378833

[8] Alhazzani W, Parhar KKS, Weatherald J, et al. Effect of awake prone positioning on endotracheal intubation in patients with COVID-19 and acute respiratory failure: a randomized clinical trial. JAMA. 2022;327(21):2104–13. 35569448 10.1001/jama.2022.7993 PMC9108999

[9] Garrett R, Shijing J, Ritwick A, et al. Smartphone-guided self-prone positioning vs usual care in nonintubated hospital ward patients with COVID-19: a pragmatic randomized clinical trial. Chest. 2022;162(4):782–91. 35597286 10.1016/j.chest.2022.05.009 PMC9116967

[10] Grasselli G, Calfee CS, Camporota L, et al. ESICM guidelines on acute respiratory distress syndrome: definition, phenotyping and respiratory support strategies. Intensive Care Med. 2023;49(7):727–59. 37326646 10.1007/s00134-023-07050-7 PMC10354163

[11] Weatherald J, Parhar KKS, Al Duhailib Z, et al. Efficacy of awake prone positioning in patients with covid-19 related hypoxemic respiratory failure: systematic review and meta analysis of randomized trials. BMJ. 2022;379: e071966. 36740866 10.1136/bmj-2022-071966 PMC9727649

[12] Mariano E, Marina B, Nora A, et al. Impact of exposure time in awake prone positioning on clinical outcomes of patients with COVID-19-related acute respiratory failure treated with high-flow nasal oxygen: a multicenter cohort study. Crit Care. 2022;26(1):16. 34996496 10.1186/s13054-021-03881-2 PMC8740872

[13] Liu L, Sun Q, Zhao H, et al. Prolonged vs shorter awake prone positioning for COVID-19 patients with acute respiratory failure: a multicenter, randomised controlled trial. Intensive Care Med. 2024;50(8):1298–309. 39088076 10.1007/s00134-024-07545-x PMC11306533

[14] Hu SL, He HL, Pan C, et al. The effect of prone positioning on mortality in patients with acute respiratory distress syndrome: a meta-analysis of randomized controlled trials. Crit Care. 2014;18(3):R109. 24887034 10.1186/cc13896 PMC4075407

[15] Ibarra-Estrada M, Li J, Pavlov I, et al. Factors for success of awake prone positioning in patients with COVID-19-induced acute hypoxemic respiratory failure: analysis of a randomized controlled trial. Crit Care. 2022;26(1):84. 35346319 10.1186/s13054-022-03950-0 PMC8958810

[16] Qin S, Chang W, Peng F, et al. Awake prone position in COVID-19-related acute respiratory failure: a meta-analysis of randomized controlled trials. BMC Pulm Med. 2023;23(1):145. 37101160 10.1186/s12890-023-02442-3 PMC10131466

[17] Qian ET, Gatto CL, Amusina O, et al. Assessment of awake prone positioning in hospitalized adults with COVID-19: a nonrandomized controlled trial. JAMA Intern Med. 2022;182(6):612–21. 35435937 10.1001/jamainternmed.2022.1070 PMC9016608

[18] Zhang W, He Y, Gu Q, et al. Optimal timing for awake prone positioning in Covid-19 patients: insights from an observational study from two centers. Int J Nurs Stud. 2024;152: 104707. 38368846 10.1016/j.ijnurstu.2024.104707

[19] Grieco DL, Delle Cese L, Menga LS, et al. Physiological effects of awake prone position in acute hypoxemic respiratory failure. Crit Care. 2023;27(1):315. 37592288 10.1186/s13054-023-04600-9 PMC10433569

[20] Johnson SA, Horton DJ, Fuller MJ, et al. Patient-directed Prone Positioning in Awake Patients with COVID-19 Requiring Hospitalization (PAPR). Ann Am Thorac Soc. 2021;18(8):1424–6. 33596394 10.1513/AnnalsATS.202011-1466RL PMC8513661

[21] Yarahmadi S, Ebrahimzadeh F, Mohamadipour F, et al. Effect of prone position on clinical outcomes of nonintubated patients with COVID-19: a randomised clinical trial. Collegian. 2023;30(3):449–56. 36591534 10.1016/j.colegn.2022.12.005 PMC9792421

[22] Luo J, Pavlov I, Tavernier E, et al. Awake prone positioning in adults with COVID-19 an individual participant data meta-analysis. JAMA Intern Med. 2025: e250011. 10.1001/jamainternmed.2025.0011 PMC11894540 40063016

[23] Ling S, Zhimin L, Zhanqi Z. How often do we need to update PEEP setting during prone positioning in ARDS? Crit Care. 2024;28(1):60. 38409024 10.1186/s13054-024-04847-w PMC10898165

[24] Sud S, Friedrich JO, Taccone P, et al. Prone ventilation reduces mortality in patients with acute respiratory failure and severe hypoxemia: systematic review and meta-analysis. Intensive Care Med. 2010;36(4):585–99. 20130832 10.1007/s00134-009-1748-1

[25] Xu Q, Wang T, Qin X, et al. Early awake prone position combined with high-flow nasal oxygen therapy in severe COVID-19: a case series. Crit Care. 2020;24(1):250. 32448330 10.1186/s13054-020-02991-7 PMC7246000

[26] Kaur R, Vines DL, Mirza S, et al. Early versus late awake prone positioning in non-intubated patients with COVID-19. Crit Care. 2021;25(1):340. 34535158 10.1186/s13054-021-03761-9 PMC8446738

[27] Davis HE, McCorkell L, Vogel JM, et al. Long COVID: major findings, mechanisms and recommendations. Nat Rev Microbiol. 2023;21(3):133–46. 36639608 10.1038/s41579-022-00846-2 PMC9839201

