# Supplementary material for: Impact of awake prone positioning duration on intubation or mortality in COVID-19 patients with acute respiratory failure: secondary analysis of a randomized clinical trial
Source: Ann Intensive Care. 2025 Jun 23;15:84. doi: 10.1186/s13613-025-01501-8 (PMC12185815; doi:10.1186/s13613-025-01501-8)
Supplement: Supplementary file 1 — Additional file 1 [file 13613_2025_1501_MOESM1_ESM.docx]

Longer duration of awake prone positioning was associated with improved outcomes among COVID-19 patients with acute respiratory failure: a secondary analysis of prolonged awake prone positioning trial

Qin Sun^1*^, Rui Zhang^1*^, Junyi Zhang^1^, Jianfeng Xie^1^, Yingzi Huang^1^, Yi Yang^1^, Haibo Qiu^1^, Ling Liu^1†^, Hui Chen^1†^, on behalf of the Chi-ARDS Net (Chinese ARDS Research Network).

[Table S1: Cox proportional hazard model to analyze the effect of daily duration of APP on the primary outcome. 2](#_Toc196983978)

[Table S2: Cox proportional hazard model with interaction between allocated to standard care group and daily duration of APP. 3](#_Toc196983979)

[Table S3: Cox proportional hazard model with interaction between allocated to standard care group and total duration of APP. 4](#_Toc196983980)

[Table S4: Cox proportional hazard model with interaction between allocated to standard care group and average duration of APP. 5](#_Toc196983981)

[Table S5: Cox proportional hazard model to analyze the effect of daily APP duration on 28-day mortality. 6](#_Toc196983982)

[Table S6: Cox regression model with Heaviside functions to analyze the effect of different exposure windows of APP on the primary outcome. 7](#_Toc196983983)

[Table S7: Cox proportional hazard model to analyze the effect of the duration of APP on the primary outcome after excluding patients who were intubated or deceased on Day 1 (N=387). 8](#_Toc196983984)

[Figure S1: Study schedule of the present study 10](#_Toc196983985)

[Figure S2: Comparison of duration of APP between patients who failed and who succeeded on high-flow nasal cannula. 11](#_Toc196983986)

[Figure S3: The effect of unmeasured confounders as quantified by E-value. 12](#_Toc196983987)

## Table S1: Cox proportional hazard model to analyze the effect of daily duration of APP on the primary outcome.

|  | HR (95% CI) | P value |
| --- | --- | --- |
| Duration of APP 8-12 h/d | Reference | Reference |
| Duration of APP < 8 h/d | 2.44 (1.21-4.92) | 0.013 |
| Duration of APP >12 h/d | 1.03 (0.51-2.10) | 0.932 |
| Age, years | 1.03 (1.01-1.06) | 0.004 |
| Sex (Female) | 1.04 (0.66-1.64) | 0.866 |
| BMI, kg/m^2^ | 0.99 (0.94-1.05) | 0.799 |
| Location at enrolment |  |  |
| Intensive care unit | Reference | -- |
| Sub intensive care unit | 0.75 (0.43-1.28) | 0.289 |
| General ward | 1.16 (0.29-4.54) | 0.836 |
| Non-invasive respiratory support^*^ | 4.11 (2.58-6.55) | <0.001 |
| SpO_2_/FiO_2_, per 10 | 0.94 (0.46-1.89) | 0.851 |
| SOFA score | 1.28 (1.16-1.40) | <0.001 |
| Allocated to standard care group | 0.90 (0.54-1.52) | 0.704 |

^*^ Non-invasive respiratory support including high-flow nasal cannula and non-invasive ventilation.

APP: Awake prone positioning; HR: Hazard ratio; CI: Confidence interval; BMI: Body mass index; SpO_2_: Peripheral oxygen saturation; FiO_2_: Fraction of inspired oxygen; SOFA: Sequential organ failure assessment.

## Table S2: Cox proportional hazard model with interaction between allocated to standard care group and daily duration of APP.

|  | HR (95% CI) | P value |
| --- | --- | --- |
| Daily duration of APP, h | 0.97 (0.88-1.08) | 0.594 |
| Age, years | 1.04 (1.01-1.06) | 0.004 |
| Sex (Female) | 1.06 (0.68-1.66) | 0.788 |
| BMI, kg/m^2^ | 0.99 (0.94-1.05) | 0.872 |
| Location at enrolment |  |  |
| Intensive care unit | Reference | -- |
| Sub intensive care unit | 0.69 (0.41-1.18) | 0.179 |
| General ward | 1.20 (0.32-4.42) | 0.788 |
| Non-invasive respiratory support^*^ | 6.04 (1.69-21.58) | 0.005 |
| SpO_2_/FiO_2_, per 10 | 0.90 (0.46-1.79) | 0.772 |
| SOFA score | 1.28 (1.17-1.40) | <0.001 |
| Allocated to standard care group | 1.76 (0.47-6.57) | 0.403 |
| Allocated to standard care group* Daily duration of APP | 0.94 (0.83-1.06) | 0.289 |

^*^ Non-invasive respiratory support including high-flow nasal cannula and non-invasive ventilation.

APP: Awake prone positioning; HR: Hazard ratio; CI: Confidence interval; BMI: Body mass index; SpO_2_: Peripheral oxygen saturation; FiO_2_: Fraction of inspired oxygen; SOFA: Sequential organ failure assessment.

## Table S3: Cox proportional hazard model with interaction between allocated to standard care group and total duration of APP.

|  | HR (95% CI) | P value |
| --- | --- | --- |
| Total duration of APP, h | 0.96 (0.95-0.97) | <0.001 |
| Age, years | 1.02 (0.99-1.04) | 0.101 |
| Sex (Female) | 1.08 (0.70-1.66) | 0.74 |
| BMI, kg/m^2^ | 0.98 (0.92-1.03) | 0.407 |
| Location at enrolment |  |  |
| Intensive care unit | Reference |  |
| Sub intensive care unit | 0.77 (0.46-1.29 | 0.321 |
| General ward | 0.85 (0.31-2.33) | 0.751 |
| Non-invasive respiratory support^*^ | 4.58 (1.68-12.49) | 0.003 |
| SpO_2_/FiO_2_, per 10 | 0.87 (0.46-1.65) | 0.673 |
| SOFA score | 1.23 (1.12-1.35) | <0.001 |
| Allocated to standard care group | 0.65 (0.32-1.35) | 0.25 |
| Allocated to standard care group*Total duration of APP | 0.98 (0.95-0.99) | 0.041 |

^*^ Non-invasive respiratory support including high-flow nasal cannula and non-invasive ventilation.

APP: Awake prone positioning; HR: Hazard ratio; CI: Confidence interval; BMI: Body mass index; SpO_2_: Peripheral oxygen saturation; FiO_2_: Fraction of inspired oxygen; SOFA: Sequential organ failure assessment.

## Table S4: Cox proportional hazard model with interaction between allocated to standard care group and average duration of APP.

|  | HR (95% CI) | P value |
| --- | --- | --- |
| Average duration of APP, h | 0.91 (0.83-1.004) | 0.061 |
| Age, years | 1.03 (1.01-1.06) | 0.009 |
| Sex (Female) | 1.06 (0.70-1.62) | 0.781 |
| BMI, kg/m^2^ | 0.99 (0.94-1.05) | 0.834 |
| Location at enrolment |  |  |
| Intensive care unit | Reference |  |
| Sub intensive care unit | 0.77 (0.46-1.31) | 0.333 |
| General ward | 1.28 (0.46-3.62) | 0.636 |
| Non-invasive respiratory support^*^ | 6.11 (2.22-16.85) | <0.001 |
| SpO_2_/FiO_2_, per 10 | 0.85 (0.45-1.62) | 0.622 |
| SOFA score | 1.27 (1.16-1.39) | <0.001 |
| Allocated to standard care group | 0.89 (0.27-2.94) | 0.843 |
| Allocated to standard care group* Average duration of APP | 0.99 (0.88-1.11) | 0.863 |

## Table S5: Cox proportional hazard model to analyze the effect of daily APP duration on 28-day mortality.

|  | HR (95% CI) | P value |
| --- | --- | --- |
| Daily duration of APP, h | 0.95 (0.91-1.003) | 0.065 |
| Age, years | 1.05 (1.02-1.08) | <0.001 |
| Sex (Female) | 1.17 (0.73-1.88) | 0.505 |
| BMI, kg/m^2^ | 0.99 (0.94-1.06) | 0.899 |
| Location at enrolment |  |  |
| Intensive care unit | Reference |  |
| Sub intensive care unit | 0.57 (0.33-0.98) | 0.042 |
| General ward | 1.03 (0.31-3.47) | 0.961 |
| Non-invasive respiratory support^*^ | 5.04 (1.59-16.01) | 0.006 |
| SpO_2_/FiO_2_, per 10 | 1.13 (0.54-2.37) | 0.748 |
| SOFA score | 1.29 (1.16-1.43) | <0.001 |
| Allocated to standard care group | 0.96 (0.56-1.62) | 0.867 |

APP: Awake prone positioning; HR: Hazard ratio; CI: Confidence interval; BMI: Body mass index; SpO_2_: Peripheral oxygen saturation; FiO_2_: Fraction of inspired oxygen; SOFA: Sequential organ failure assessment.

## Table S6: Cox regression model with Heaviside functions to analyze the effect of different exposure windows of APP on the primary outcome.

|  | Day 1-7 | | Day 1-3 | | Day 4-5 | | Day 6-7 | |
| --- | --- | --- | --- | --- | --- | --- | --- | --- |
|  | HR (95% CI) | P value | HR (95% CI) | P value | HR (95% CI) | P value | HR (95% CI) | P value |
| Daily duration of APP, h* | 0.93 (0.88-0.98) | 0.006 | 0.93 (0.88-0.98) | 0.016 | 0.96 (0.86-1.07) | 0.42 | 0.93 (0.85-1.008) | 0.077 |

*Adjusting for age, sex, body-mass index, respiratory support of high-flow nasal cannula or non-invasive ventilation, SpO_2_/FiO_2_, sequential organ failure assessment score and treatment assignment.

## Table S7: Cox proportional hazard model to analyze the effect of the duration of APP on the primary outcome after excluding patients who were intubated or deceased on Day 1 (N=387).

|  | HR (95% CI) | P value |
| --- | --- | --- |
| Duration of APP, hours | 0.94 (0.88-0.99) | 0.029 |
| Age, years | 1.03 (1.01-1.06) | 0.012 |
| Sex (Female) | 0.90 (0.55-1.47) | 0.673 |
| BMI, kg/m^2^ | 1.01 (0.95-1.07) | 0.771 |
| Location at enrolment |  |  |
| Intensive care unit | Reference |  |
| Sub intensive care unit | 0.68 (0.37-1.25) | 0.218 |
| General ward | 1.46 (0.27-7.95) | 0.659 |
| Non-invasive respiratory support^*^ | 6.10 (1.21-30.67) | 0.028 |
| SpO_2_/FiO_2_, per 10 | 0.89 (0.42-1.89) | 0.755 |
| SOFA score | 1.34 (1.21-1.49) | <0.001 |
| Allocated to standard care group | 0.94 (0.53-1.65) | 0.831 |

^*^ Non-invasive respiratory support including high-flow nasal cannula and non-invasive ventilation.

APP: Awake prone positioning; HR: Hazard ratio; CI: Confidence interval; BMI: Body mass index; SpO_2_: Peripheral oxygen saturation; FiO_2_: Fraction of inspired oxygen; SOFA: Sequential organ failure assessment.

Table S8: Cox proportional hazard model to analyze the effect of the duration of APP on early and late APP failure.

|  | Early APP failure (<7 days) | | Late APP failure (≥7 days) | |
| --- | --- | --- | --- | --- |
|  | HR (95% CI) | P value | HR (95% CI) | P value |
| Duration of APP, hours | 0.94 (0.88-0.99) | 0.025 | 0.88 (0.79-0.98) | 0.024 |
| Age, years | 1.04 (1.01-1.08) | 0.008 | 1.01 (0.97-1.04) | 0.75 |
| Sex (Female) | 1.23 (0.74-2.05) | 0.418 | 0.37 (0.14-0.99) | 0.048 |
| BMI, kg/m^2^ | 1.01 (0.94-1.08) | 0.816 | 1.02 (0.92-1.12) | 0.736 |
| Location at enrolment |  |  |  |  |
| Intensive care unit | Reference | -- | Reference | -- |
| Sub intensive care unit | 0.59 (0.31-1.10) | 0.098 | 0.49 (0.19-1.26) | 0.141 |
| General ward | 0.95 (0.25-3.59) | 0,936 | 0.46 (0.13-1.62) | 0.23 |
| Non-invasive respiratory support^*^ | 7.87 (2.14-28.95) | 0.001 | 1.85 (0.59-5.80) | 0.294 |
| SpO_2_/FiO_2_, per 10 | 1.16 (0.48-2.81) | 0.739 | 0.85 (0.23-3.02) | 0.799 |
| SOFA score | 1.31 (1.18-1.45) | <0.001 | 1.44 (1.21-1.72) | <0.001 |
| Allocated to standard care group | 1.10 (0.61-1.97) | 0.756 | 0.61 (0.29-1.27) | 0.186 |

^*^ Non-invasive respiratory support including high-flow nasal cannula and non-invasive ventilation.

APP: Awake prone positioning; HR: Hazard ratio; CI: Confidence interval; BMI: Body mass index; SpO_2_: Peripheral oxygen saturation; FiO_2_: Fraction of inspired oxygen; SOFA: Sequential organ failure assessment.


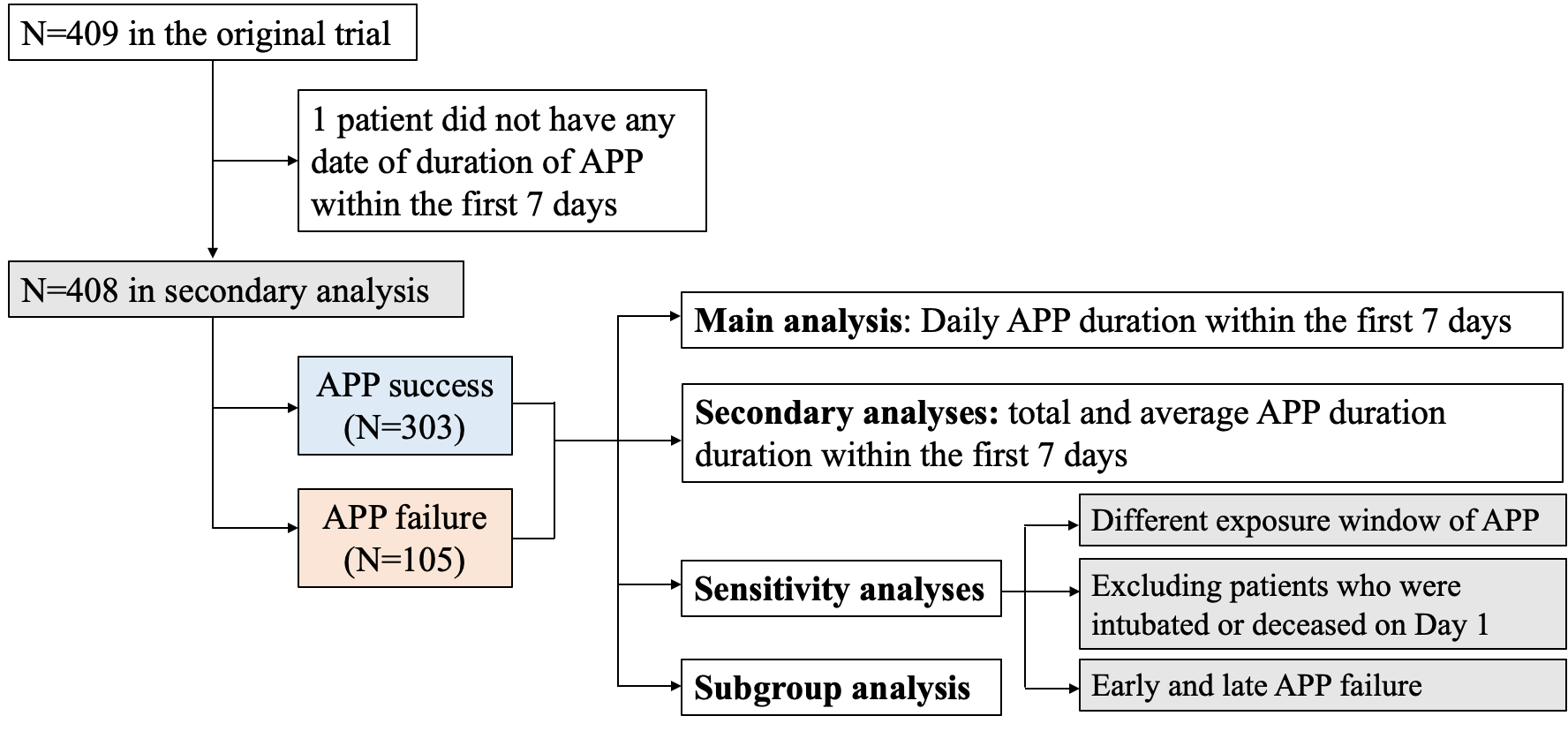


## Figure S1: Study schedule of the present study


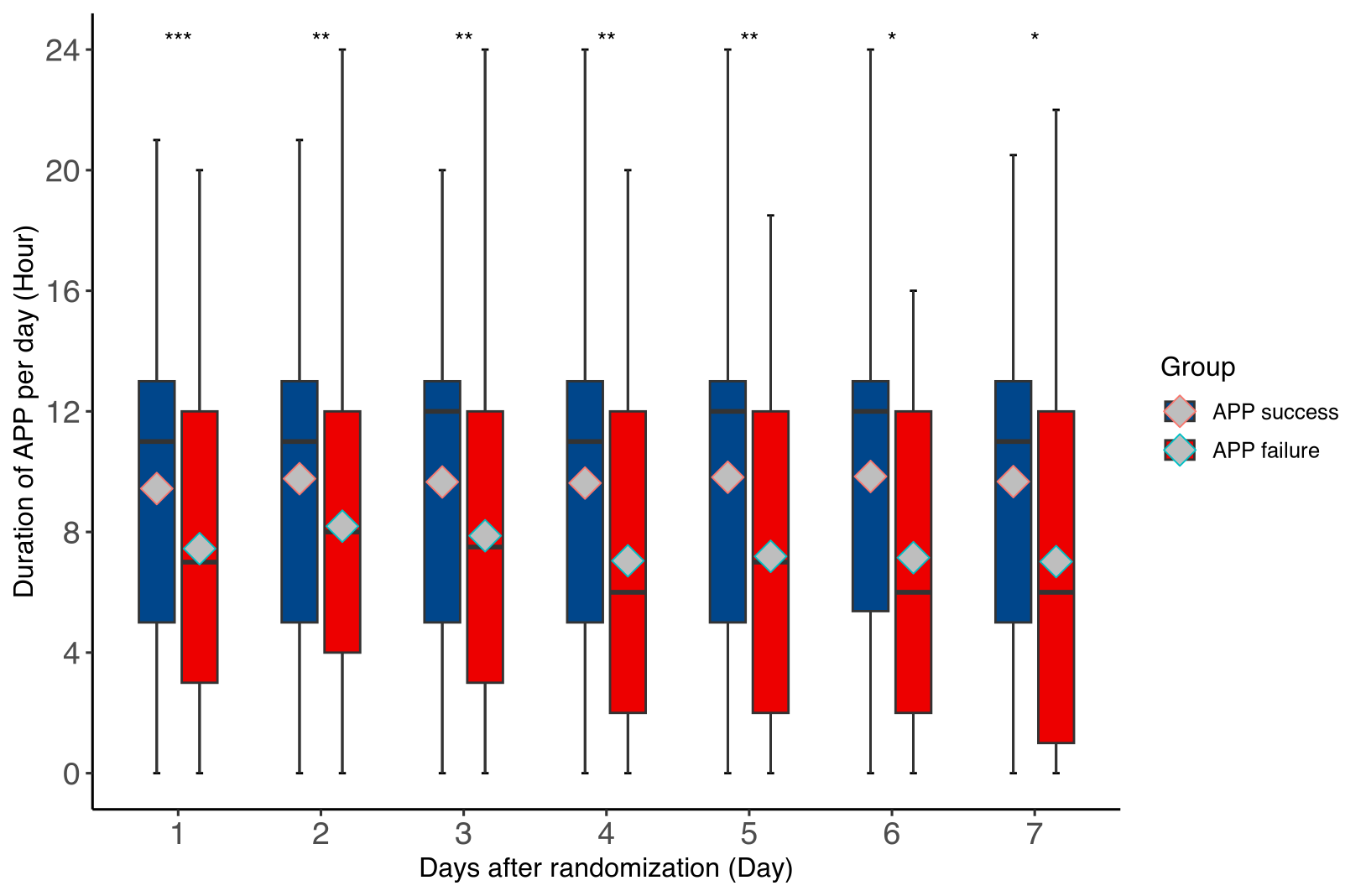


## Figure S2: Comparison of duration of APP between patients who failed and who succeeded on high-flow nasal cannula.

^*^P<0.05, ^**^P<0.01, ^***^P<0.001.


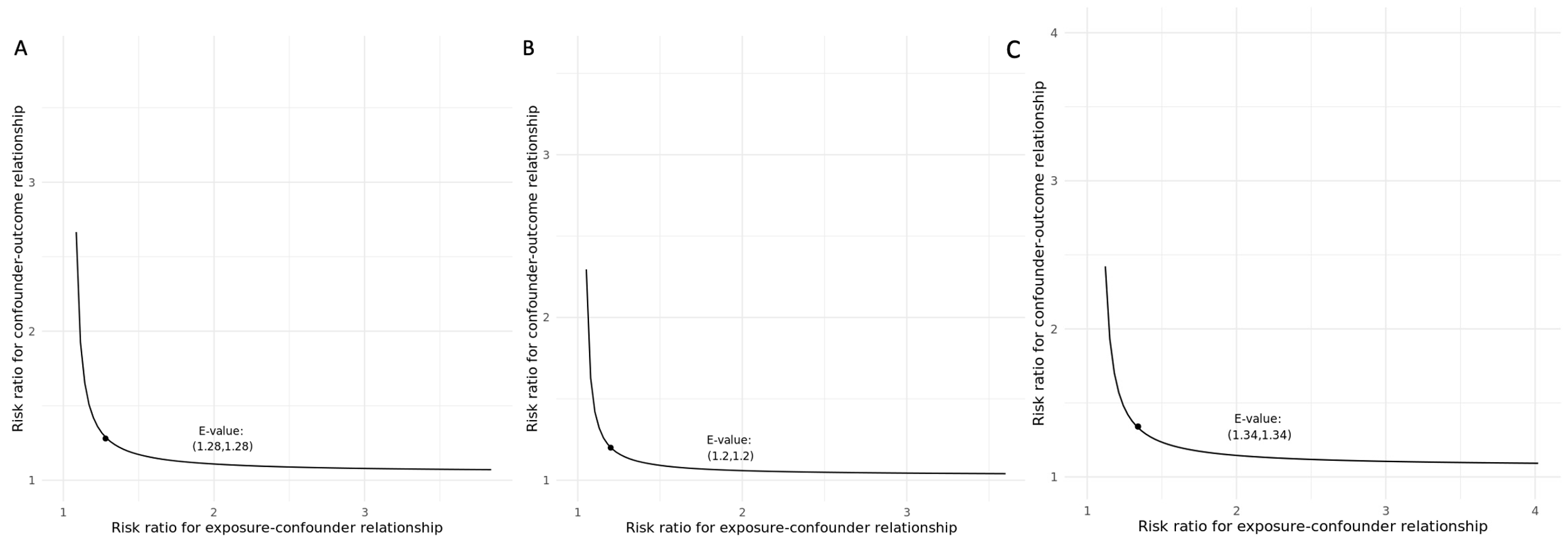


## Figure S3: The effect of unmeasured confounders as quantified by E-value.

A: adjusting for the daily duration of the APP; B: adjusting for the total duration of the APP; C: adjusting for the average daily APP duration.
